# The clinical characteristics and etiological study of nonalcoholic fatty liver disease in Chinese women with PCOS

**Published:** 2013-09

**Authors:** Zhongyu Qu, Yanhui Zhu, Jingjing Jiang, Yuhua Shi, Zijiang Chen

**Affiliations:** 1 *Division of Ultrasonography, Shandong Provincial Hospital, 324 Jingwu Road, Jinan, Shandong 250021, China.*; 2 *Medical Informatics Center, Peking University, 38 Xueyuan Road, Beijing 100191, China.*; 3 *Reproductive Medicine Center, Shandong University, 157 Jingliu Road, Jinan, Shandong 250021, China.*

**Keywords:** Polycystic ovary syndrome, Infertility, Nonalcoholic fatty liver disease.

## Abstract

**Background:** Polycystic ovary syndrome (PCOS) is highly associated with non-alcoholic fatty liver disease (NAFLD). There are extensive ethnic differences in the clinical manifestations, pathological changes, and ovarian changes in women with PCOS.

**Objective: **To investigate the prevalence and clinical characteristics of NAFLD in Chinese women with PCOS.

**Materials and Methods:** Non-pregnant women with PCOS (N= 602) and matched controls without PCOS (N=588) were recruited. Basal endocrine, oral glucose tolerance test, insulin release level, lipid level, blood pressure, and body mass index (BMI) were measured. Liver biochemical and B-hepatitis and C-hepatitis indices were determined.

**Results: **NAFLD was significantly more prevalent in women with PCOS than controls (32.9% vs. 18.5%) and included 113 (57.1%) mild, 75 (37.8%) moderate and 10 (5.1%) severe cases. Luteinizing hormone was significantly lower in PCOS women with NAFLD than without NAFLD. In the PCOS group, NAFLD prevalence and severity increased with BMI. The liver index was significantly higher (p<0.001), and the quantitative insulin sensitivity check index and high density lipoprotein cholesterol were significantly lower (p<0.001) in the PCOS group than controls. Insulin resistance, abdominal obesity, diabetes mellitus, abnormal glucose tolerance, liver dysfunction, dyslipidemia, hypertension, and metabolic syndrome were significantly more prevalent in the NAFLD group than controls.

**Conclusion:** Chinese women with PCOS have a high prevalence of mostly mild and moderate NAFLD, not significantly associated with hyperandrogenism that increased significantly with BMI. Insulin resistance and metabolic abnormalities are important factors associated with NAFLD. Chinese women with BMI ≥24 kg/m^2^ should be screened for NAFLD.

## Introduction

In 1935, Stein and Leventhal published the first description of what is now called polycystic ovary syndrome (PCOS) (1). The condition is well recognized as having a major lifetime effect on reproductive abilities and metabolism of affected women. Insight into the pathogenesis and treatment of PCOS has increased substantially in the past decades. PCOS is a syndrome of ovarian dysfunction; its cardinal features are hyperandrogenism and polycystic ovary morphology (2). 

The clinical manifestations include menstrual irregularities, signs of androgen excess, and obesity. PCOS is associated with an increased risk for type 2 diabetes mellitus. The differential diagnosis includes congenital adrenal hyperplasia, Cushing’s syndrome, and androgen-secreting tumours (3). Non-alcoholic fatty liver disease (NAFLD) refers to the presence of hepatic steatosis not associated with a significant intake of ethanol that is histologically identical to alcoholic liver disease after exclusion of nutritional disorders, drugs, and diseases known to cause secondary fatty liver disease (4). 

NAFLD encompasses a spectrum of liver diseases including simple steatosis, nonalcoholic steatohepatitis, and nonalcoholic steatohepatitis-associated cirrhosis (5-7). NAFLD may be categorized as primary or secondary depending on the underlying pathogenesis. Primary NAFLD occurs most commonly and is associated with an insulin-resistant state, such as diabetes and obesity. Other conditions associated with insulin resistance, such as PCOS and hypopituitarism, have also been described in association with NAFLD (8). 

Insulin resistance can promote lipolysis of peripheral adipose tissue, which in turn increases free fatty acid influx into the liver. Hyperinsulinemia and hyperglycemia also promote *de novo* lipogenesis and indirect inhibition of free fatty acid oxidation (9, 10). 

PCOS is highly associated with NAFLD because both diseases are associated with insulin resistance. The prevalence of NAFLD in women with PCOS was 55% (48/88) in the U.S., and 41.5% (17/41) in Chile (6, 7). There are extensive ethnic differences in the clinical manifestations, pathological changes, and ovarian changes in women with PCOS. 

The purpose of the current research is to study the prevalence and clinical features of Chinese women with PCOS, to analysis risk factors for the prevalence of NAFLD in women with PCOS, the effect of BMI on hyperandrogenism on the prevalence of NAFLD in women with PCOS; and the screening index for NAFLD.

## Materials and methods

**Patients**

A total of 602 non-pregnant women with PCOS presenting at the Reproductive Medicine Centre, Shandong University, between May 2008 and December 2010, were enrolled in this cross sectional study. Another group of 588 non-pregnant tubal obstructed women of similar age and body mass index (BMI) were recruited to serve as a control group. All patients enrolled with written consent. 

All of them were ethnic Han Chinese, who lived in the area of Shandong Province, in the middle-eastern region of China. The patients enrolled in the control group were those with normal menstruation and endocrinology who underwent in vitro fertilization embryo transfer therapy because of ovarian obstruction. The 588 women in the control group were excluded from a diagnosis of PCOS by clinical findings and laboratory examinations. There was no significant difference in the BMI between the 602 women with PCOS and the 588 women in the control group.

The diagnostic standard for PCOS was the Rotterdam standard, revised by the European Society for Human Reproduction and Embryology and the American Society for Reproductive Medicine in 2003 (11). The Rotterdam consensus expanded the diagnostic criteria to include at least two of the following three features: clinical and/or biochemical hyperandrogenism, oligoanovulation, and polycystic ovary diagnosed by ultrasonography after excluding other endocrinopathies such as Cushing syndrome, hyperprolactinemia, congenital adrenal hyperplasia, and ovulation disorders  ( 4, 11). Women with a history of acute viral hepatitis, haemochromatosis, autoimmune liver disease, or other diseases were excluded.

**Clinical, anthropometric, and laboratory data**

The protocol was adapted from Cerda *et al* and included a pre-coded questionnaire with socioeconomic data, ethnicity, medical history including diagnosis of hypertension and diabetes, a history of alcohol and tobacco consumption, a physical examination, and blood tests (7). Excessive alcohol consumption was defined as more than 20 g of alcohol daily. 

Anthropometric measurements included height, weight, waist circumference, hip circumference, BMI, and waist-to-hip ratio. The Ferriman Gallwey scoring system was used to evaluate the hirsutism. Obesity was defined as BMI ≥28 kg/m^2^ (12-14). Fatty liver was diagnosed with abdominal ultrasound using standardized criteria. Abdominal ultrasound was performed in all subjects with the same equipment (GE Volusion 730 Pro, Milwaukee, Wisconsin, United States) and doctor, who was unaware of the clinical and laboratory results. HOMA-IR can be reliably used in large-scale or epidemiological studies in which only a fasting blood sample is available to assess insulin sensitivity (15). 

HOMA-IR was calculated with the following formula: fasting serum insulin (μU/ml) × fasting plasma glucose (mmol/L) /22.5 (16, 17). Using this method, high HOMA-IR scores denote low insulin sensitivity (insulin resistance). QUICKI is derived using the inverse of the sum of the logarithms of the fasting insulin and fasting glucose as follows:

1/(log [fasting insulin µU/mL] + log [fasting glucose mg/dL]) (18, 19).

Blood samples were taken at 8 am after fasting during the 2-4 days of menstruation; follicle stimulating hormone (FSH), luteinizing hormone (LH), prolactin, estradial, testosterone, dehydroepiandrosterone (DHES), and insulin were tested by chemiluminescence; fasting blood glucose was tested by the glucose oxidase method, using the Beckman instruments. Autonomous biochemical analyzers were used to assess liver function, total cholesterol, high density lipoprotein cholesterol (HDL-C), low density lipoprotein cholesterol (LDL-C), and triglycerides. 

**Quality control**

Clinicians in the Reproductive Medicine Centre, Shandong University, and researchers in the Medical Informatics Centre, Peking University, conducted the epidemiological investigation. The research protocol was approved by the Ethics Committee of the Reproductive Medicine Centre, Shandong University. Five percent of the questionnaires, blood samples drew from median cubital vein, and ultrasonography were randomly sampled, and the Kappa analysis indicated that the diagnostic results had good consistency.

**Diagnostic standards**

The 1999 WHO standards were used to diagnose increased fasting blood glucose levels, abnormal glucose tolerance, diabetes mellitus, and hypertension. The diagnostic standard for hyperinsulinemia was fasting blood glucose ≥0.015 IU/ml and/or glucose loading insulin ≥0.080 IU/ml. Women were diagnosed with hyperandrogenemia when any of the signs of hyperandrogenism, characterized by hirsutism, androgenic alopecia, or repeated attacks of acne and virilism, were present. The biochemical index was serum testosterone >2.08 nmol/L. 

NAFLD diagnosis required confirmation of hepatic steatosis based on imaging studies but excluded subjects who regularly consumed >20gr ethanol per day and those with fatty liver resulting from diseases like viral hepatitis, total parenteral nutrition, autoimmune responses, metabolic or hereditary factors, and drugs or toxins (20). NAFLD patients could have elevated serum transaminase and clinical manifestations including hepatomegaly, general malaise, abdominal discomfort, vague right upper quadrant abdominal pain, nausea, or other nonspecific symptoms. NAFLD severity was graded as mild, moderate, and severe based on echogenicity of the liver parenchyma and the visualization of intrahepatic vessels and the diaphragm (6, 21, 22). 

**Statistical analysis**

The clinical characteristics and laboratory measurements of women with PCOS and the control group were analyzed and continuous variables were presented as means±SD. Qualitative variables were presented as percentages. Comparisons between groups were performed with the Student’s* t*-test for parametric data. Homogeneity of sample variances were performed by the homogeneity test of variances, and Chi-square test for proportions. 

Data were analyzed with SPSS for Windows statistical package version 13.0 (SPSS Inc., Chicago, IL, USA). A p-value of <0.01 was considered statistically significant. 

## Results

**The prevalence of NAFLD in women with PCOS**

Of 602 women with PCOS, 198 were diagnosed with NAFLD. The prevalence rate was 32.9%, among which 113 cases (57.1%) were mild, 75 cases (37.8%) were moderate, and 10 cases (5.1%) were severe. Of 588 women in the control group, 109 were diagnosed with NAFLD. The prevalence rate was 18.5%, among which 69 cases (63.3%) were mild, 35 cases (32.1%) were moderate, and 5 cases (4.6%) were severe. The prevalence of NAFLD in the group with PCOS was significantly higher than in the control group (p<0.001).

**Analysis of risk factors for having both PCOS and NAFLD**

The 602 women with PCOS were divided into those with (NAFLD^+^ group, 198 cases) and without (NAFLD^-^ group, 404 cases) NAFLD. The one-way analysis of variance (ANOVA) suggested that fasting blood glucose (p<0.001), 2-hour blood glucose after oral glucose tolerance test (p<0.001), 5-times insulin level (serum fasting insulin 30, 60, 120 and 180 min insulin) (p<0.001), HOMA-IR (p<0.001), ALT (p=0.004), AST (p<0.001), total cholesterol (p<0.001), triglycerides (p<0.001), LDL-C (p<0.001), BMI (p<0.001), and the WRH (p<0.001) of the NAFLD^+^ group were significantly higher compared with the NAFLD^-^ group, and that QUICKI and HDL-C levels in the NAFLD^+^ group were significantly lower compared with the NAFLD^-^ group (p<0.001) (Table I). 

**Logistic regression analysis**

In the logistic regression analysis, NAFLD was considered the dependent variable and age, BMI, WHR, total cholesterol, triglyceride, and HOMA-IR were considered independent variables. The forward stepwise method was adopted with an entry standard of 0.05, and elimination standard of 0.10. The order entered into the equation was BMI, triglycerides, WHR, and HOMA-IR; other factors did not enter the equation (Table II). 

As seen in Table II, the prevalence of NAFLD in women with PCOS was positively related to BMI, triglycerides, WHR, and HOMA-IR, and the regression coefficient test was statistically significant. Age, FSH, LH, prolactin, testosterone, estradiol, DHES, and total cholesterol levels were not significantly correlated with the prevalence of NAFLD in women with PCOS. 

**The effect of** **BMI on** **the prevalence of** **NAFLD in women with PCOS**

The prevalence and severity of NAFLD increased significantly with the increase of BMI in women with PCOS. In women with PCOS and BMI ≥28 kg/m^2^, the prevalence of NAFLD reached up to 83.8%; 39.4% had mild cases compared with only a 3.6% prevalence of mild NAFLD in women with PCOS and 18.5≤BMI<24 kg/m^2^, as shown in table III (p<0.001).

**Comparison of the prevalence of associated disease or abnormalities between women with PCOS and NAFLD and women with PCOS but without NAFLD**

The prevalence of insulin resistance, abdominal obesity, diabetes mellitus, abnormal glucose tolerance, liver dysfunction, dyslipidemia, hypertension, and metabolic syndrome in women with PCOS and NAFLD was significantly higher than in women with PCOS but without NAFLD (p<0.001; Chi-square test) (Table IV).

**The effect of** **hyperandrogenism on** **the prevalence of** **NAFLD in** **women with PCOS** The women were divided into 3 groups; those with normal androgen group (testosterone <1.56 nmol/L), those with mild hyperandrogenism (testosterone 1.56-2.08 nmol/L), and those with hyperandrogenism (testosterone >2.08 nmol/L). The prevalence of NAFLD was not significantly different among these three groups (Table V). 

**Table I:** Comparison of women with PCOS and NAFLD (NAFLD^+^) with those who did not have NAFLD (NAFLD^-^)

**Variable**	**NAFLD** ^+^ ** (n=198** **）**	**NAFLD** ^-^ ** (n=404** **）**	**p-** **value**
Age (yr)	28.71 ± 3.53	28.14 ± 3.85	0.082
FSH (IU/L)	6.21 ± 1.35	6.48 ± 1.68	0.051
LH (IU/L)	10.13 ± 4.52	11.57 ± 5.05	0.001
Prolactin (IU/L)	0.367 ± 0.161	0,388 ± 0.163	0.142
Estradiol (nmol/L)	0.149 ± 0.055	0.159 ± 0.054	0.126
Testosterone (nmol/L)	2.13 ± 1.06	2.06 ± 0.89	0.416
DHES(μg/dL)	216.23 ± 76.71	217.14 ±71.84	0.886
Insulin 0 min (IU/L)	0.018 ± 0.006	0.010 ± 0.005	<0.001
Insulin 30 min (IU/L)	0.101 ± 0.063	0.070 ± 0.043	<0.001
Insulin 60 min (IU/L)	0.132 ± 0.072	0.0828 ± 0.055	<0.001
Insulin 120 min (IU/L)	0.116 ± 0.076	0.059 ± 0.045	<0.001
Insulin 180 min (IU/L)	0.041 ± 0.037	0.023 ± 0.021	<0.001
Glucose 0 min (mmol/L)	5.55 ± 1.17	4.93 ± 0.73	<0.001
Glucose 30 min (mmol/L)	8.99 ± 1.94	8.03 ± 1.46	<0.001
Glucose 60 min (mmol/L)	9.51 ± 2.85	7.79 ± 1.95	<0.001
Glucose 120 min (mmol/L)	7.03 ± 1.60	6.01 ± 1.50	<0.001
Glucose 180 min (mmol/L)	5.14 ± 1.85	4.76 ± 1.32	<0.001
ALT (IU/L)	31.65 ± 20.87	18.36 ± 11.99	0.004
GGT (IU/L)	25.37 ± 14.20	16.32 ± 9.46	<0.001
AST (IU/L)	23.38 ± 12.92	19.79 ± 8.84	<0.001
Total protein (g/L)	71.45 ± 3.78	71.26 ± 5.22	<0.001
Albumin (g/L)	44.13 ± 3.78	44.24 ± 3.73	0.690
Total cholesterol (mmol/L)	4.95 ± 0.96	4.48 ± 0.84	0.746
HDL-C (mmol/L)	1.05 ± 0.25	1.30 ± 0.32	<0.001
LDL-C (mmol/L)	2.65 ± 0.76	2.29 ± 0.66	<0.001
Triglycerides (mmol/L)	1.92 ± 1.07	1.12 ± 0.70	<0.001
Waist circumference (cm)	96.81 ± 7.75	80.2 ± 7.56	<0.001
Systolic blood pressure (mmHg)	123.52 ± 14.10	113.45 ± 11.59	<0.001
Diastolic blood pressure (mmHg)	78.93 ± 10.68	69.73 ± 9.47	<0.001
BMI (kg/m^2^)	29.10 ± 3.22	23.06 ± 3.09	<0.001
WHR	0.92 ± 0.05	0.87 ± 0.05	<0.001
QUICKI	0.31 ± 0.02	0.35 ± 0.03	<0.001
HOMA-IR	4.38 ± 1.73	2.22 ± 1.19	<0.001

FSH: follicle stimulation hormone; LH: luteinizing hormone; DHES: dehydroepiandrosterone; ALT: alanine aminotransferase; GGT: Gamma-glutamyl transferase; AST: the aspartate aminotransferase; HDL-C: high density lipoprotein cholesterol; LDL-C: low density lipoprotein cholesterol; BMI: body mass index; WHR: waist hip ratio; QUICKI: quantitative insulin sensitivity check index; HOMA-IR: homeostasis model assessment of insulin resistance, NAFLD: non-alcoholic fatty liver disease; PCOS: polycystic ovary syndrome.

Comparisons between groups were performed with the Student’s* t*-test. A p-value of <0.01 was considered statistically significant.

**Table II:** The correlation between risk factors and the prevalence of NAFLD in women with PCOS

**Index**	**Regression coefficient**	**Standard error**	**Wald-** **chi-square value**	**p-** **value**
BMI	0.24	0.07	11.55	0.001
Triglycerides	0.44	0.16	7.33	0.007
WHR	11.62	4.44	6.84	0.009
Waist circumference	0.18	0.04	20.62	<0.001
HOMA-IR	0.54	0.13	18.09	<0.001
Constant	﹣36.03	4.15	75.55	<0.001

BMI: body mass index; WHR: waist-hip ratio; HOMA-IR: homeostasis model assessment of insulin resistance, NAFLD: non-alcoholic fatty liver disease; PCOS: polycystic ovary syndrome.

Analysis were performed with the logistic regression. A p-value of <0.01 was considered statistically significant.

**Table III:** Relationship between BMI and NAFLD in women with PCOS

**BMI (kg/m2)**	**Cases**	**NAFLD-**			**NAFLD+** ** (mild)**			**NAFLD+** ** (moderate)**			**NAFLD+** ** (severe)**		
		**Cases**	**%**	**p-value**	**Cases**	**%**	**p-value**	**Cases**	**%**	**p-value**	**Cases**	**%**	**p-value**
<18.5	22	22	100.0	0.9062*	0	0.0	0.3713*	0	0	-	0	0	-
18.5- <24	247	238	96.4	1.0000*	9	3.6	1.0000*	0	0	1.0000*	0	0	1.0000*
24 - <28	191	121	63.4	0.0044*	48	25.1	<0.0001*	22	11.5	<0.0001*	0	0	-
≥28	142	23	16.2	<0.0001*	56	39.4	<0.0001*	53	37.3	<0.0001*	10	7.1	<0.0001*

BMI: body mass index; NAFLD: non-alcoholic fatty liver disease; PCOS: polycystic ovary syndrome.

Analysis were performed with the Chi-square test. A p-value of <0.01 was considered statistically significant.

* Compared with the BMI 18.5≤ to <24 kg/m2 group.

**Table IV:** Comparison of the prevalence of associated diseases and abnormalities in women with (NAFLD^+^) or without NAFLD (NAFLD^-^)

**Variable**	**NAFLD+ ** **(** **n=198** **）**	**NAFLD- (n=404** **）**	**p-value**
IR (n=368)	187 (94.4%)	181 (44.8%)	<0.001*
Abdominal obesity (n=245)	156 (78.8%)	89 (22.0%)	<0.001*
Diabetes mellitus (n=33)	28 (14.1%)	5 (1.2%)	<0.001*
Abnormal glucose tolerance (n=89)	56 (28.3%)	33 (8.2%)	<0.001*
Liver dysfunction (n=86)	56 (28.3%)	30 (7.4%)	<0.001*
Dyslipidemia (n=146)	84 (42.4%)	62 (15.3%)	<0.001*
Hypertension (n=29)	21 (10.1%)	8 (2.0%)	<0.001*
Metabolic syndrome (n=177)	163 (82.3%)	14 (3.5%)	<0.001*

NAFLD: non-alcoholic fatty liver disease; IR: insulin resistance.

Metabolic syndrome refers to the standard revised at the 2003 Rotterdam conference (The Rotterdam ESHRE/ASRM -Sponsored PCOS Work shop Group, 2004a).

Analysis were performed with the Chi-square test. A p-value of <0.01 was considered statistically significant.

* Compared with NAFLD^-^ group.

**Table V:** The relationship between hyperandrogenism and PCOS in women with NALFD (NAFLD^+^) or without NAFLD (NAFLD^-^)

**Groups (nmol/L)**	**Cases**	**NAFLD** ^+^	**NAFLD** ^-^
Testosterone <1.56	217	77 (37.0%)	140 (63.0%)
Testosterone 1.56-2.08	114	44 (31.4%)	70 (68.6%)
Testosterone >2.08	271	114 (36.1%)	157 (63.9%)

NAFLD: non-alcoholic fatty liver disease; PCOS: polycystic ovary syndrome.

Analysis were performed with the Chi-square test. A p-value of <0.01 was considered statistically significant.

Differences among groups were not statistically significant (p=0.332).

## Discussion

In our study, the prevalence of NAFLD in Chinese women with PCOS was 32.9%, which was significantly higher than the 18.5% prevalence in the control group. However, this prevalence rate is lower than the 55% (48/88) prevalence of NAFLD reported in American women with PCOS, and the 41.5% (17/41) prevalence of NAFLD in Chilean women with PCOS (6, 7). 

Most women with PCOS who also had NAFLD had mild (57.1%) or moderate (37.8%) type. The difference in prevalence rates in different regions of the world relates to heredity, ethnicity, and lifestyle modifications like moderate weight loss and exercise (23-25). In Cerda *et al *pioneering study in Chilean women, women with PCOS who also had NAFLD had higher mean BMI (34.51±7.02 kg/m^2^) compared with the group without NAFLD (27.48±5.59 kg/m^2^). However, they failed to stratify the BMI groups to show the correlation between the prevalence of NAFLD and BMI (7). Gambarin-Gelwan *et al* concluded that high BMI and insulin resistance appeared to be important factors associated with the prevalence of NAFLD in women with PCOS (6). 

This group also failed to stratify BMI to show the correlation between the prevalence of NAFLD and BMI. We also found that women with PCOS who had NAFLD had higher mean BMI (29.10±3.22 kg/m^2^) compared with women with PCOS who did not have NAFLD (mean BMI 23.06±3.09 kg/m^2^). Interestingly, the BMI of Chinese women with PCOS, both with and without NAFLD, was lower than that of the Chilean women (7). In our study, the logistic regression analysis showed that the prevalence of NAFLD in women with PCOS was positively related to BMI, triglycerides, WHR, waist circumference, and HOMA-IR. 

The prevalence and severity of NAFLD increased with the increase of BMI, and severe NAFLD did not occur in women with a low BMI (BMI <24). Women with both PCOS and NAFLD had greater insulin resistance and were more likely to have metabolic syndrome than women with PCOS who did not have NAFLD (6, 7, 26). DeUgarte *et al* found that the prevalence of insulin resistance was 64% in women with PCOS. Women with insulin resistance were more clinically affected (27). Marchesini *et al* found that insulin resistance was significantly associated with high liver enzyme levels, and might contribute to hepatocellular necrosis *via* liver fat accumulation (28). 

Accordingly, most researchers consider that the high prevalence of PCOS might be induced by insulin resistance. The results of our study support this hypothesis; the one way ANOVA of our study indicates that HOMA-IR, 5-times insulin level in OGTT, and glucose level in the group of women with PCOS and NAFLD was significantly higher than in women with PCOS who did not have NAFLD, whereas the QUICKI in the group of women with PCOS and NAFLD was significantly lower than that in women with PCOS who did not have NAFLD. 

The logistic regression analysis indicated that HOMA-IR was positively related to the prevalence of NAFLD in women with PCOS. HOMA-IR is a sensitive index for evaluating insulin resistance. When HOMA-IR is higher, insulin resistance is also higher, and the prevalence of NAFLD in women with PCOS is higher. Paradis *et al. *also support that the hyperglycemia and insulin are the key factors in the progression of liver injury, the mechanism of which is the up-regulation of connective tissue growth factor (29).

Although insulin resistance is a common abnormality in women with PCOS, it does not seem to be a universal feature (24). In our study, LH levels were significantly lower in women with PCOS who also had NAFLD than in those who did not have NAFLD, whereas BMI in those with PCOS and NAFLD was significantly higher than in those with PCOS who did not have NAFLD. This can be explained by Pagán *et al* study, reporting that the pituitary response to a weight-based dose of GnRH is inversely related to BMI in women with PCOS, and that there was an inverse relationship between LH and BMI in women with PCOS (30).

Regarding other hormones that affect the prevalence of NAFLD in women with PCOS, our results indicated that there are no significant differences in testosterone or DHES levels between women with both PCOS and NAFLD and those with PCOS who do not have NAFLD (Table I). There were no significant differences in the prevalence of NAFLD in women with PCOS among the different testosterone groups (Table V). Our results suggest that hyperandrogenemia has no significant effect on the prevalence of NAFLD in women with PCOS. This conclusion contradicts Vassilatou *et al* study, who found clinically significant NAFLD only in women with PCOS who had increased total androgen levels (31). 

The cause and effect relationship between PCOS and NAFLD or other diseases is complex. We noticed a review searched Medline (1985-2010) for peer-reviewed articles related to PCOS and NAFLD (32). The articles were summarized and grouped according to different sections defining interactions between PCOS with metabolic syndrome and NAFLD, and found many articles that concluded that NAFLD is the hepatic manifestation of metabolic syndrome. PCOS is often considered an ovarian manifestation of metabolic syndrome. 

Both of these conditions can co-exist and may respond to similar therapeutic strategies. Nader *et al.’s* study also concluded that both PCOS and non-alcoholic steatohepatitis are associated with metabolic syndrome and insulin resistance, except for a few case reports (33). In summary, the prevalence of NAFLD in Chinese women with PCOS is high, and mostly of mild and moderate types. The prevalence and severity of NAFLD is positively related to BMI. Those who are overweight or have abdominal obesity should be checked for NAFLD and encouraged to change their life style. The prevalence of NAFLD is closely related to insulin resistance and abnormal metabolism, but was not related to hyperandrogenism.
